# Association of elevated serum soluble CD226 levels with the disease activity and flares of systemic lupus erythematosus

**DOI:** 10.1038/s41598-021-95711-2

**Published:** 2021-08-09

**Authors:** Miki Nakano, Masahiro Ayano, Kazuo Kushimoto, Shotaro Kawano, Kazuhiko Higashioka, Shoichiro Inokuchi, Hiroki Mitoma, Yasutaka Kimoto, Mitsuteru Akahoshi, Nobuyuki Ono, Yojiro Arinobu, Koichi Akashi, Takahiko Horiuchi, Hiroaki Niiro

**Affiliations:** 1 Department of Medicine and Biosystemic Science, Kyushu University Graduate School of Medical Sciences, 3-1-1 Maidashi, Higashi-ku, Fukuoka, 812-8582 Japan; 2 Department of Cancer Stem Cell Research, Kyushu University Graduate School of Medical Sciences, 3-1-1 Maidashi, Higashi-ku, Fukuoka, 812-8582 Japan; 3 Department of Internal Medicine, Kyushu University Beppu Hospital, 4546 Tsurumibaru, Tsurumi, Beppu, 874-0838 Japan; 4 Department of Medical Education, Kyushu University Graduate School of Medical Sciences, 3-1-1 Maidashi, Higashi-ku, Fukuoka, 812-8582 Japan

**Keywords:** Autoimmunity, Autoimmune diseases

## Abstract

CD226 is an activating receptor expressed on the cell surface of natural killer cells and T cells. Although CD226 polymorphism is known to be involved in systemic lupus erythematosus (SLE), the involvement of soluble CD226 (sCD226) in SLE is still unknown. In the present study, we measured serum sCD226 levels using an enzyme-linked immunosorbent assay in 58 SLE patients and 33 healthy controls (HCs) and evaluated their associations with SLE Disease Activity Index 2000 (SLEDAI-2K), clinical manifestations, laboratory data, and the cumulative probability of flare. Serum sCD226 levels showed no significant differences between SLE patients and HCs. However, sCD226 levels were significantly elevated in active SLE patients with a SLEDAI-2K score of ≥ 20 compared with HCs. In SLE patients, sCD226 levels were significantly correlated with SLEDAI-2K scores and anti-dsDNA antibody titers. Moreover, the cumulative probability of flare was markedly higher in patients with high sCD226 than in those with low sCD226. In patients with neuropsychiatric involvement, sCD226 levels were elevated and reflected neuropsychiatric disease activity. These findings indicate that serum sCD226 levels are associated with disease activity and flares of SLE. Thus, it may be a useful biomarker for SLE, and its monitoring allows for more precise SLE management.

## Introduction

Systemic lupus erythematosus (SLE) is a multi-systemic autoimmune disease with diverse clinical manifestations^1,2^, commonly with renal and neuropsychiatric involvement, and shows variable severities^2,3^. Because the clinical course of SLE varies and flares occur several times, it is important to diagnose clinical manifestations and monitor the disease activity^4^. Biomarkers are valuable for assessing disease activities and predicting flares, but useful biomarkers have not been established yet.

The pathogenesis of SLE is multifactorial and includes genetic factors^2,5–7^. Genome-wide association studies have reported an association between nonsynonymous rs763361 polymorphism in CD226 and SLE in multiple ancestries^8–11^. CD226 is a transmembrane glycoprotein mainly expressed on T cells and natural killer (NK) cells, which acts as an activating receptor and mediates cytotoxicity^12–14^. CD226 plays an important role in the immune system, and a previous study showed that the proportion of CD226 on NK cells was decreased in active SLE patients and that CD226^+^ NK cells may be involved in the immunopathogenesis of SLE^15^.

A soluble form of CD226 (sCD226), which is shed from the membrane type of CD226 (mCD226) in human serum, has been identified. The utility of sCD226 as a biomarker has been reported in acute graft-versus-host disease (aGVHD)^16,17^ and some types of cancers^18–20^. As for autoimmune diseases, a more recent study found that serum sCD226 levels were associated with disease activity in rheumatoid arthritis (RA)^21^. Although several findings suggest that CD226 is involved in the pathogenesis of SLE^8–11,15^, the association between sCD226 and SLE is still unknown.

This study aimed to reveal the association of sCD226 with SLE by measuring serum sCD226 levels using an enzyme-linked immunosorbent assay (ELISA) in SLE patients, as well as by evaluating the associations between sCD226 levels and the disease activity, clinical manifestations, and flares of SLE.

## Results

### Serum sCD226 levels are increased in active SLE patients and reflect disease activity

**Figure 1:**
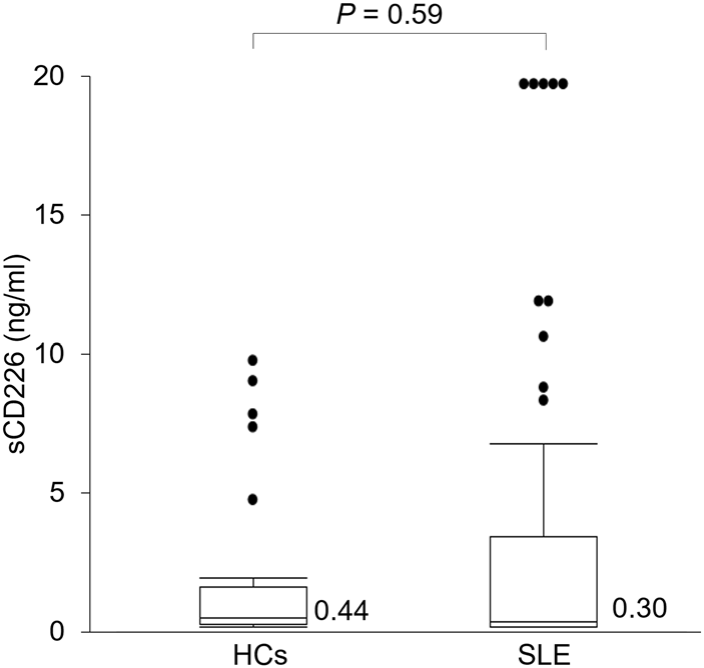
Serum sCD226 levels in SLE patients and HCs. Serum sCD226 levels were compared between SLE patients and HCs. Data are shown as box plots. The boxes represent the upper and lower IQRs; lines inside the boxes represent the median; whiskers represent 1.5 times the upper and lower IQRs; points outside the whiskers represent outliers. Statistical differences among groups were evaluated using the Mann–Whitney *U* test. *sCD226* soluble CD226, *SLE* systemic lupus erythematosus, *HCs* healthy controls, *IQRs* interquartile ranges.

**Figure 2:**
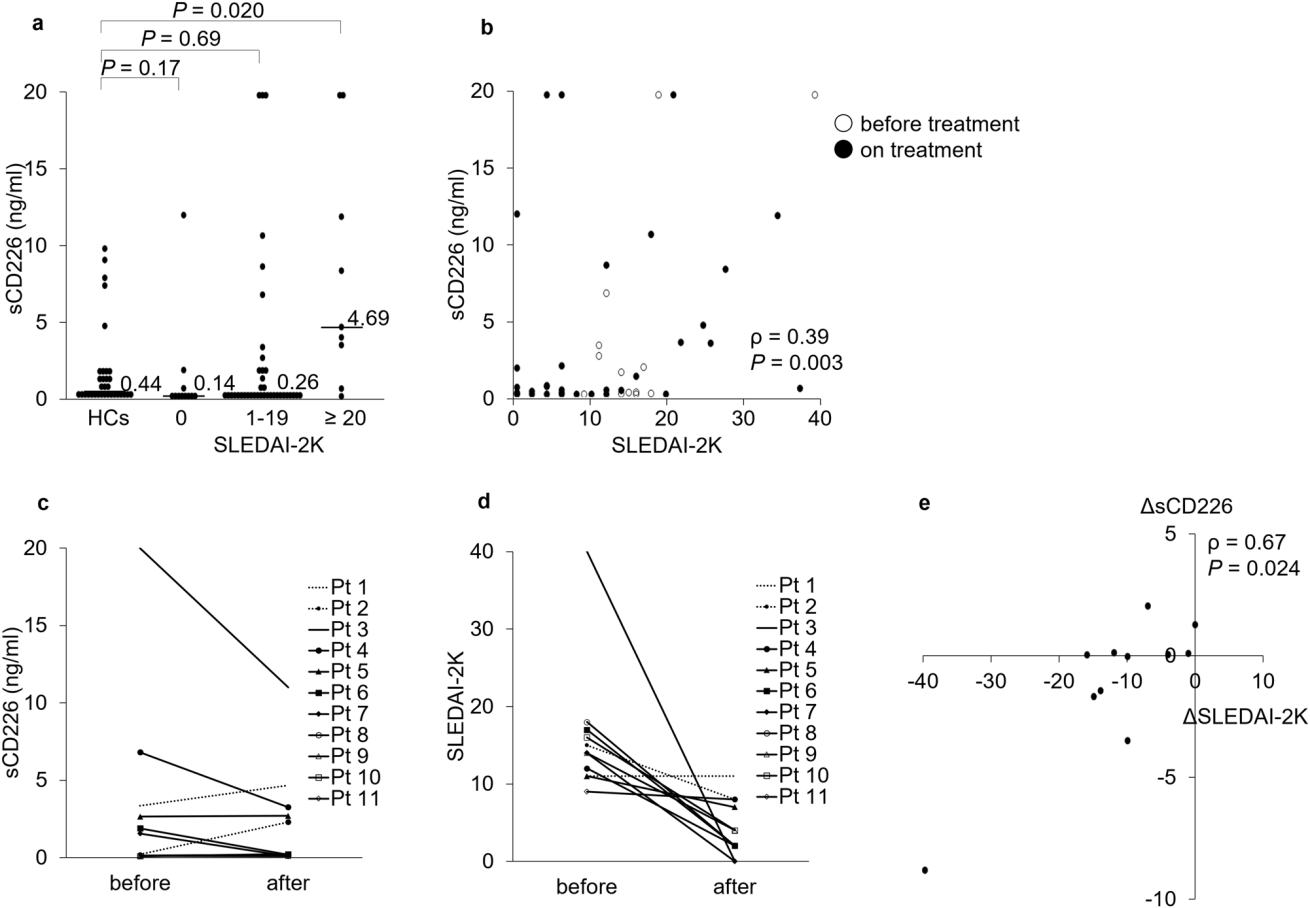
Associations between serum sCD226 levels and SLEDAI-2K scores. (**a**) Serum sCD226 levels were compared between SLE patients with SLEDAI-2K scores of 0, 1–19, and ≥ 20, as well as HCs. (**b**) Correlations between serum sCD226 levels and SLEDAI-2K scores in SLE patients. (**c**) Serum sCD226 levels before and after treatment in 11 SLE patients. (**d**) SLEDAI-2K scores before and after treatment in 11 SLE patients. (**e**) Correlations between the ΔsCD226 and the ΔSLEDAI-2K scores in SLE patients. Each data point represents a single subject. Horizontal lines show the median. Statistical differences among groups were evaluated using the Steel test, setting HCs as a control. Correlation analyses were evaluated using Spearman’s rank correlation. *sCD226* soluble CD226, *SLE* systemic lupus erythematosus, *SLEDAI-2K* SLE Disease Activity Index 2000, *HCs* healthy controls, *ΔsCD226* changes in sCD226, *ΔSLEDAI-2K* changes in SLEDAI-2K.

To study the association between sCD226 and SLE, we first measured serum sCD226 levels using ELISA in 58 SLE patients (mean age, 41.0 years; 53 females) and 33 healthy controls (HCs) (mean age, 36.2 years; 28 females). No significant differences were found between SLE patients and HCs in terms of age and gender. The baseline characteristics of the SLE patients are shown in Table 1. There were 42 patients receiving treatment. Thus, we first confirmed that serum sCD226 levels were almost the same between SLE patients with medication, such as corticosteroids and immunosuppressive agents, and those without medication and were not significantly correlated with prednisolone equivalent dose (see Supplementary Fig. S1). Although there was no significant difference between SLE patients and HCs regarding median levels of serum sCD226, these still had a wide range of values and the SLE patients had higher values than had HCs (Fig. 1). Because of this, we classified the SLE patients into three groups on the basis of SLE Disease Activity Index 2000 (SLEDAI-2K) scores, and then compared sCD226 levels between HCs and these groups. Serum sCD226 levels were found to be significantly elevated in active SLE patients than in other SLE patients and HCs (Fig. 2a). Similarly, serum sCD226 levels were increased in an active SLE patient receiving no treatment than in other 15 SLE patients receiving no treatment [20.0 ng/ml vs 0.22 ng/ml (0.10–2.65); *P* = 0.15].

**Table 1:** Baseline characteristics of patients with SLE.

Characteristics	
Age, mean (SD), years	41.0 (14.4)
Female, *n* (%)	53 (91)
Disease duration, median [IQR], years	8.0 [1–17]
Previous lupus nephritis, *n* (%)	12 (21)
Previous NPSLE, *n* (%)	10 (17)
**SLE disease activity**	
SLEDAI-2K score, median [IQR]	10.5 [2.0–16.3]
SLEDAI-2K ≥ 20, *n* (%)	9 (16)
**Clinical manifestations, *n* (%)**	
Renal	28 (48)
Mucocutaneous	23 (40)
Hematological	14 (24)
Musculoskeletal	11 (19)
Neuropsychiatric	10 (17)
Cardiorespiratory	9 (16)
Gastrointestinal	2 (3)
Ophthalmic	0 (0)
**Increased anti-dsDNA antibodies, *n* (%)**	30 (52)
Anti-dsDNA antibody titer, median [IQR]	43.4 [24.5–279.1]
Low complement, *n* (%)	37 (64)
C3, median [IQR], mg/dl	71 [48–89.5]
C4, median [IQR], mg/dl	11.5 [7–16]
No medication, *n* (%)	16 (28)
**Corticosteroid use, *n* (%)**	41 (71)
Prednisolone equivalent dose, median [IQR], mg/day	9 [5–22.5]
Hydroxychloroquine use, *n* (%)	5 (9)
Immunosuppressive agent use, *n* (%)	29 (50)

*SLE* systemic lupus erythematosus, *IQR* interquartile range, *NPSLE* neuropsychiatric systemic lupus erythematosus, *SLEDAI-2K* systemic lupus erythematosus disease activity index in 2000.

We next studied the relationship between sCD226 levels and SLE disease activity. Serum sCD226 levels had a significantly positive correlation with SLEDAI-2K (ρ = 0.39; *P* = 0.003) (Fig. 2b). When compared with conventional biomarkers, sCD226 levels were significantly correlated with anti-dsDNA antibody titers (ρ = 0.28; *P* = 0.035) and inversely correlated with serum levels of C3 (ρ =  − 0.17; *P* = 0.19) and C4 (ρ =  − 0.20; *P* = 0.14) (see Supplementary Fig. S2). In comparison with laboratory findings, serum sCD226 levels had no obvious correlation to white blood cell count, platelet count, erythrocyte sedimentation rate, and C-reactive protein (see Supplementary Fig. S3).

Furthermore, we also examined sCD226 levels in 11 SLE patients before and after treatment. Among the 11 SLE patients, serum sCD226 levels were decreased or remained low after treatment in 9 SLE patients with improvement in SLEDAI-2K scores. In contrast, serum sCD226 levels were increased in two SLE patients: one with no improvement in SLEDAI-2K scores and the other with newly neuropsychiatric manifestation after treatment (Fig. 2c,d). The changes in sCD226 had a significant correlation with the changes in SLEDAI-2K scores (ρ = 0.67; *P* = 0.024) (Fig. 2e).

### sCD226 levels can predict disease flare

**Figure 3:**
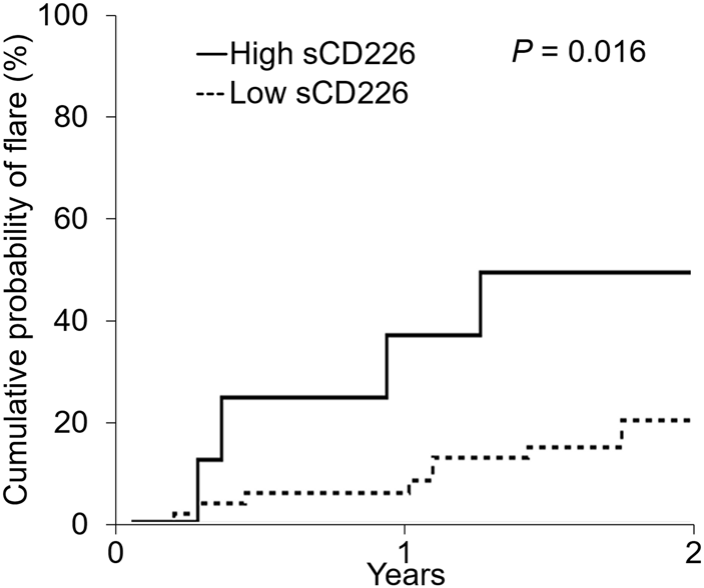
Kaplan–Meier analysis for cumulative probability of flares for patients with different levels of sCD226. Curves were compared using log-rank tests. *sCD226* soluble CD226.

In this study, flares occurred in 15 out of 58 SLE patients examined. Because the highest sCD226 level in HCs was 10.0 ng/ml, we defined this as the cut-off value for classifying SLE patients as having high and low sCD226 levels. The cumulative probability of flare for patients with high sCD226 (Fig. 3, shown in solid lines) was significantly higher than that for those with low sCD226 (Fig. 3, shown in dashed lines) (*P* = 0.016).

### sCD226 levels are increased in SLE patients with neuropsychiatric manifestation and reflect neuropsychiatric disease activity

We then assessed the association between sCD226 levels and clinical manifestations of SLE. Serum sCD226 levels were elevated in patients with mucocutaneous, hematological, musculoskeletal, and/or neuropsychiatric manifestations classified using the British Isles Lupus Assessment Group (BILAG) 2004 index (Table 2). In clinical descriptors of SLEDAI-2K, serum sCD226 levels were significantly increased in patients with psychosis, visual disturbance, arthritis, myositis, rash, and/or mucosal ulcers (see Supplementary Table S1).

**Table 2:** Serum sCD226 levels in SLE patients with each clinical manifestation.

Clinical manifestations	*n* (%)	Median sCD226 levels [IQR], ng/ml		*P*-value
		Presence	Absence	
Renal	28 (48)	0.30 [0.10–4.40]	0.30 [0.10–2.15]	0.863
Mucocutaneous	23 (40)	1.56 [0.20–8.68]	0.15 [0.10–1.29]	0.008
Hematological	14 (24)	3.07 [0.19–13.98]	0.20 [0.10–1.49]	0.041
Musculoskeletal	11 (19)	3.36 [0.20–20.0]	0.22 [0.10–1.84]	0.007
Neuropsychiatric	10 (17)	1.43 [0.32–8.47]	0.21 [0.10–2.48]	0.067
Cardiorespiratory	9 (16)	3.49 [0.16–14.2]	0.24 [0.10–1.94]	0.146

Serum sCD226 levels were compared between SLE patients with each clinical manifestation and those without. The table lists the clinical manifestation that occurred in more than 10% of patients. Statistical differences among groups were evaluated using the Mann–Whitney *U* test.

*sCD226* soluble CD226, *SLE* systemic lupus erythematosus, *IQR* interquartile range.

**Figure 4:**
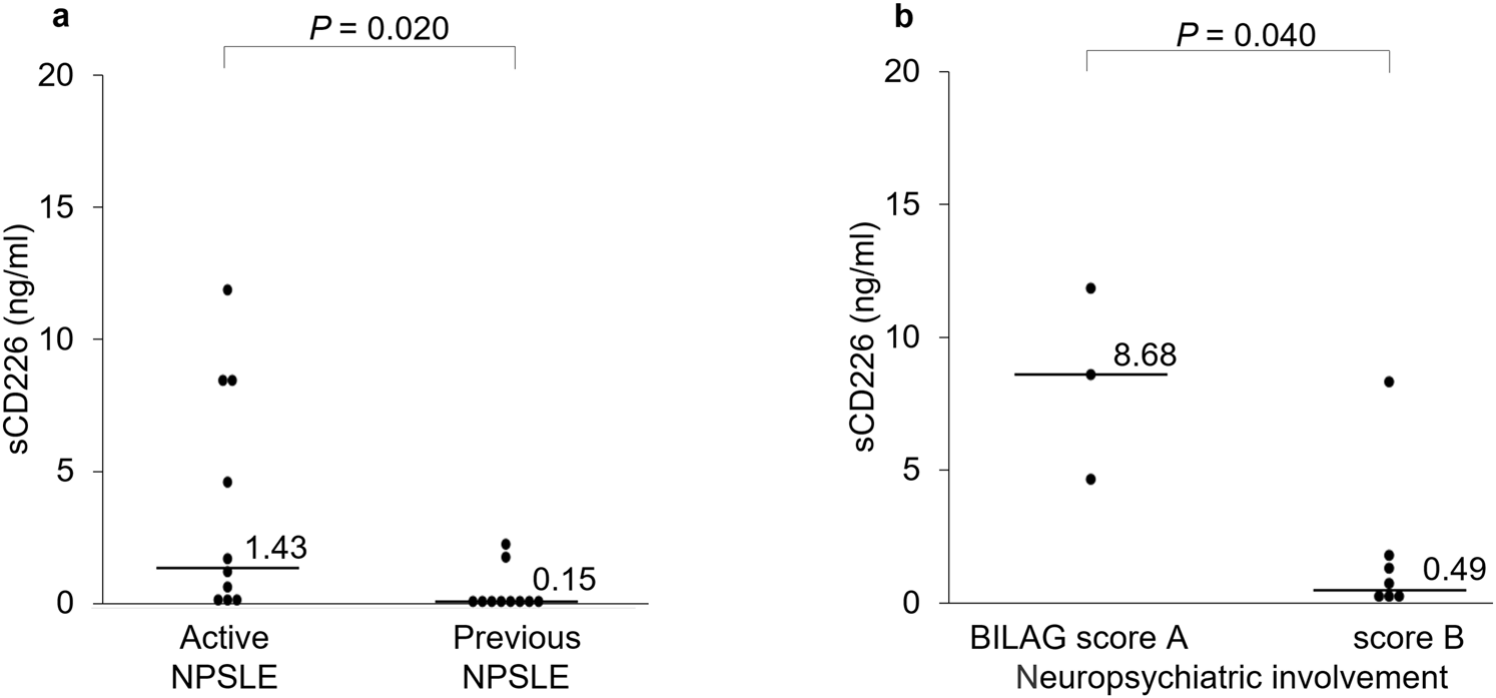
Serum sCD226 levels in SLE patients with neuropsychiatric manifestation. (**a**) Serum sCD226 levels were compared between active NPSLE patients and previous NPSLE patients. (**b**) Serum sCD226 levels were compared between neuropsychiatric SLE patients with BILAG category A and those with BILAG category B. Each data point represents a single subject. Horizontal lines show the median. Statistical differences among groups were evaluated using the Mann–Whitney *U* test. *sCD226* soluble CD226, *SLE* Systemic lupus erythematosus, *NPSLE* neuropsychiatric systemic lupus erythematosus, *BILAG* British Isles Lupus Assessment Group.

Because neuropsychiatric systemic lupus erythematosus (NPSLE) is a major vital organ manifestation of SLE^3^, we further studied the relationships between sCD226 levels and neuropsychiatric manifestation. Serum sCD226 levels were elevated in patients with active neuropsychiatric manifestation (Table 2). Serum sCD226 levels were also significantly higher in active NPSLE patients than in previous NPSLE patients (Fig. 4a). Moreover, among active NPSLE patients, sCD226 levels were significantly higher in patients with BILAG category A, which is defined as severe disease activity, than in those with BILAG category B (Fig. 4b).

## Discussion

In this study, we demonstrated that serum sCD226 levels were significantly increased in active SLE patients and were associated with disease activity and neuropsychiatric manifestation. We also showed that SLE patients with high sCD226 levels had a high probability of experiencing disease flares.

CD226 is an immunoglobulin superfamily expressed on the cell membrane of NK cells, T cells, B cells, monocytes, and platelets^12–14^. A soluble form of CD226 has been identified and was reported to be shed from the cell membrane maybe by a certain protease^16^. CD226 is a costimulatory adhesion molecule involved in certain immune functions such as mediating cytotoxic signals^12–14^, the ligands of which are CD112 and CD155^13^. This costimulatory molecule has a paired receptor, T cell immunoreceptor with immunoglobulin and immunoreceptor tyrosine-based inhibitory motif domain (TIGIT), which is a coinhibitory receptor that inhibits the interaction between CD155 and CD226, in turn also inhibits the activation of T cells and NK cells^22,23^. On the basis of the results of genome-wide association studies and functional analyses of mCD226 on T cells or NK cells, CD226 is thought to play an important role in the pathogenesis of autoimmune diseases such as SLE^8–11,15^, RA^11,24–27^, and systemic sclerosis^28–30^. However, the utility of sCD226 as a biomarker and its functions in these diseases remain unknown.

In this study, we aimed to investigate the association between sCD226 and SLE, and found that serum sCD226 levels were significantly elevated in active SLE patients than in HCs and were associated with disease activity. Moreover, we also found that the cumulative probability of flare was significantly higher in patients with high sCD226 than in patients with low sCD226, indicating that sCD226 may be predictive of flares. According to the 2019 EULAR recommendation, SLE treatment should aim for remission or low disease activity and prevention of flares^4^. Although many useful biomarkers for monitoring disease activity have been reported^31,32^, no proper biomarkers for predicting flares were found. In our study, serum sCD226 levels were associated with not only disease activity but also with SLE flares. Therefore, sCD226 may be a useful biomarker for SLE.

In this study, we further investigated the association between sCD226 levels and neuropsychiatric manifestation, which is a frequent major organ manifestation in SLE^3,4^. Diverse neuropsychiatric symptoms make it difficult to distinguish from other diseases and hinder a proper diagnosis^3^. Additionally, its management is challenging because of the variable severity^3^. Useful biomarkers for both diagnosing and monitoring disease activity are required to manage NPSLE properly, but none have been established yet^33,34^. Although cerebrospinal fluid (CSF) tests from lumbar puncture are valuable for the exclusion of infectious diseases and some CSF biomarkers, such as anti-ribosomal P protein antibodies, IgG index, and IL-6, may be useful for assessing disease activity, the procedure is invasive and difficult to repeat for monitoring^3,33^. In our study, serum sCD226 levels were elevated in patients with neuropsychiatric involvement and reflected neuropsychiatric disease activity. The findings indicate that serum sCD226 may be a useful biomarker for both diagnosing and monitoring neuropsychiatric manifestation of SLE.

Regarding the function of sCD226 in cancer, several studies reported that sCD226 may block the cytotoxicity of NK cells by blocking CD155 or CD112^18^, and that sCD226 could directly inhibit the proliferation of cancer cells in vitro^19^. In aGVHD, Kanaya et al. explained that binding of sCD226 to CD155 may cancel the inhibitory signals by TIGIT in T cells^16^. In mouse SLE models, treatment with TIGIT-Ig fusion protein reduced autoantibody production and improved survival rate^35^. These findings indicate the binding of sCD226 to CD155 may cancel the inhibitory signals by TIGIT in SLE as well; this interaction is likely involved in the pathogenesis of SLE. Although we showed the possible utility of sCD226 as a biomarker for SLE in this study, we did not study the immune functions of sCD226 in SLE. Therefore, further analyses are required to reveal the functions of sCD226 in SLE.

This study had some limitations. First, our study had a small sample size and was conducted at a single center. This study needs to be replicated with a larger sample size in a multicenter setting. Second, the functions of sCD226 are still unknown; further analyses are required to reveal this. Lastly, our study was a retrospective study. We could not measure sCD226 levels longitudinally in all SLE patients, and changes in the levels at several points or over a short period are not apparent. To ensure the association of sCD226 levels with the disease activity and prognosis of SLE, a prospective study with longitudinal assessments should be performed.

In conclusion, we showed that serum sCD226 levels were elevated in active SLE patients and were associated with disease activity and prognosis. Serum sCD226 may be a useful biomarker for SLE, and its monitoring allows for more precise SLE management.

## Methods

### Study population

We studied 58 Japanese patients who were treated for SLE at the Kyushu University Hospital between 2014 and 2020. We enrolled patients who met at least four of the American College of Rheumatology revised criteria for SLE^36^ and had no other autoimmune disease, infection, or cancer. Many of these patients were treated with corticosteroids, hydroxychloroquine, and immunosuppressive drugs, either as monotherapy or in combination. Among these 58 SLE patients, we were able to assess 11 patients both before and after treatment. We studied 33 HCs as well.

This study was approved by the ethics committee of Kyushu University Hospital (approval number 2019-481) in accordance with the Helsinki Declaration. All participants gave written informed consent.

### Data collection

We obtained the following information from the medical records of the patients: demographic data, clinical manifestations, laboratory findings, and medications at baseline and after treatment. Disease activity was evaluated using SLEDAI-2K^37^, with active SLE defined as having a SLEDAI-2K score of ≥ 20^38^. Clinical manifestations were classified using the BILAG 2004 index^39^, and patients with BILAG category A or category B at the time of sCD226 level measurement were defined as those with each clinical manifestation. Neuropsychiatric disease activity was assessed by BILAG 2004 index^39^. A flare was defined as a measurable increase in disease activity usually leading to a change of treatment^4,40^.

### Enzyme-linked immunosorbent assay

Serum sCD226 levels were measured via sandwich ELISA, in accordance with a previous report^16^. In summary, 96-well plates were coated with purified anti-human CD226 (DNAM-1) antibody (TX25; BioLegend, San Diego, CA, USA) (8 μg/ml, 100 μl/well) for 2 h at room temperature and then washed with washing buffer (0.05% Tween 20). The plates were blocked using a blocking buffer (1% BSA in PBS, 100 μl/well) for 2 h at room temperature and then washed. Recombinant human DNAM-1/CD226 Fc chimera protein (as a standard) (R&D Systems, Minneapolis, MN, USA) and serum samples were added at 100 μl/well and incubated overnight at 4 °C. The plates were washed and then incubated with human DNAM-1/CD226 biotinylated antibody (R&D Systems) (0.6 μg/ml, 100 μl/well) for 1 h at room temperature. After washing, streptavidin–horseradish peroxidase (R&D Systems) (1:200 in a washing buffer, 100 μl/well) was added and incubated for 30 min at room temperature. The plates were washed and then reacted with 3,3′,5,5′-tetramethylbenzidine substrate reagent set (BD Biosciences, San Jose, CA, USA) (100 μl/well) for 20 min at room temperature. The reaction was stopped by H_2_SO_4_ (2 N) (50 μl/well), and then absorbance was measured at 450 nm using a microtiter plate reader (Thermo Fisher Scientific, Waltham, MA, USA). All values were determined in duplicate. The assay range was 0.1–20.0 ng/ml.

### Statistical analysis

Results are expressed as the median and interquartile range unless otherwise stated. Comparisons between two groups were done using the Student’s t-test for normally distributed continuous variables or using the Mann–Whitney *U* test for non-normally distributed variables. When making multiple group comparisons, the Steel test was used for non-normally distributed variables, setting HCs as a control. The correlations between two continuous variables were analyzed using Spearman’s rank correlation. Flaring episodes were represented via the Kaplan–Meier method and compared using log-rank tests. All tests were two-tailed and *P*-values < 0.05 were considered statistically significant. All analyses were performed using the JMP software, version 15 (SAS Institute, Cary, NC, USA).

## Supplementary Information

Supplementary Information.

## Data Availability

All data generated or analyzed during this study are included in this published article and its Supplementary Information files.
