# Supplementary material for: Association of elevated serum soluble CD226 levels with the disease activity and flares of systemic lupus erythematosus
Source: Sci Rep. 2021 Aug 9;11:16162. doi: 10.1038/s41598-021-95711-2 (PMC8352936; doi:10.1038/s41598-021-95711-2)
Supplement: Supplementary file 1 — Supplementary Information. [file 41598_2021_95711_MOESM1_ESM.pdf]

Title:

Association of elevated serum soluble CD226 levels with the disease activity and flares of systemic lupus erythematosus

Authors:

Miki Nakano,[1] Masahiro Ayano,[1 2 *] Kazuo Kushimoto,[1] Shotaro Kawano,[1] Kazuhiko Higashioka,[1] Shoichiro Inokuchi,[1] Hiroki Mitoma,[1] Yasutaka Kimoto,[3] Mitsuteru Akahoshi,[1] Nobuyuki Ono, [1] Yojiro Arinobu,[1] Koichi Akashi,[1] Takahiko Horiuchi,[3] Hiroaki Niiro[4]

Author affiliations:

[1] Department of Medicine and Biosystemic Science, Kyushu University Graduate School of Medical Sciences, 3-1-1 Maidashi, Higashi-ku, Fukuoka 812-8582, Japan

[2] Department of Cancer Stem Cell Research, Kyushu University Graduate School of Medical Sciences, 3-1-1 Maidashi, Higashi-ku, Fukuoka 812-8582, Japan

[3] Department of Internal Medicine, Kyushu University Beppu Hospital, 4546 Tsurumibaru, Tsurumi, Beppu 874-0838, Japan

[4] Department of Medical Education, Kyushu University Graduate School of Medical Sciences, 3-1-1 Maidashi, Higashi-ku, Fukuoka 812-8582, Japan

* ayano.masahiro.811@m.kyushu-u.ac.jp

Supplementary Figure S1

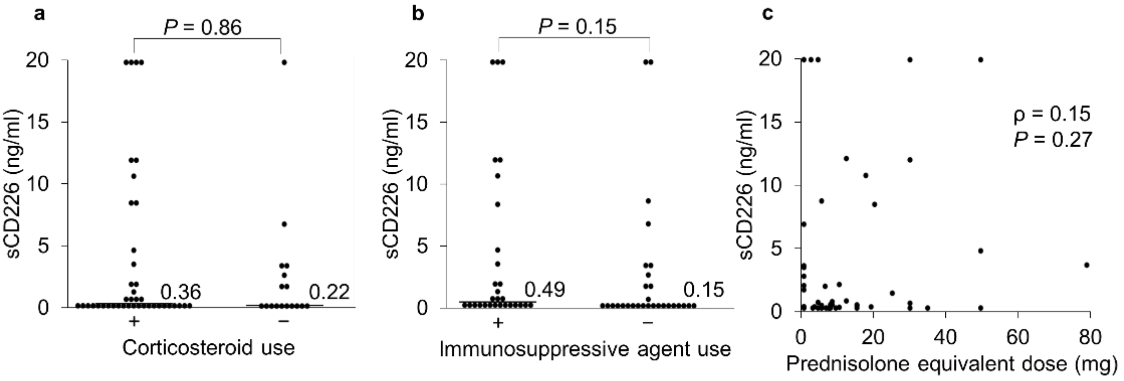

Supplementary Figure S1 Associations between serum sCD226 levels and medications (a) Serum sCD226 levels were compared between patients with corticosteroids and those without corticosteroids. (b) Serum sCD226 levels were compared between patients with immunosuppressive agents and those without immunosuppressive agents. (c) Correlations between serum sCD226 levels and prednisolone equivalent dose. Each data point represents a single subject. Horizontal lines show the median. Statistical differences among groups were evaluated using the Mann–Whitney U test. Correlation analyses were done using Spearman's rank correlation. sCD226: soluble CD226.

Supplementary Figure S2

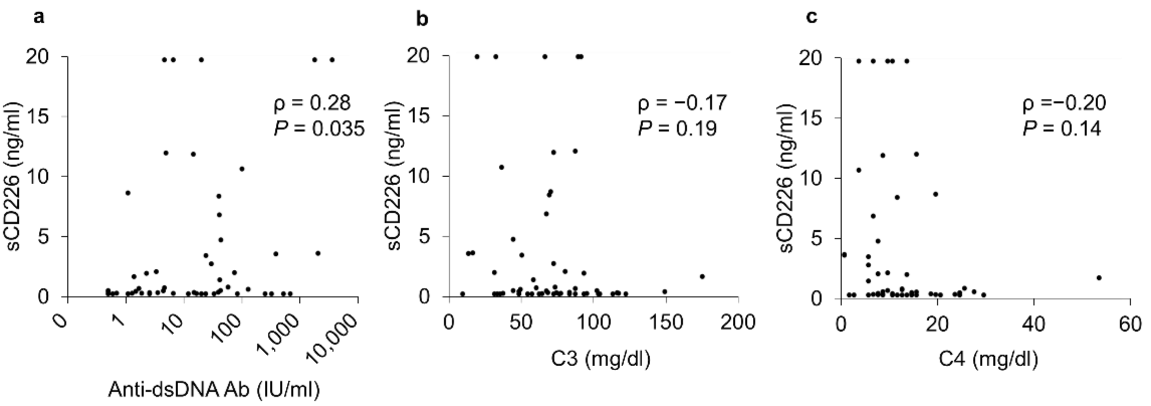

Supplementary Figure S2 Associations between serum sCD226 levels and conventional biomarkers

Correlations between serum sCD226 levels and anti-dsDNA antibody (Ab) titers (a), serum C3 (b), and C4 (c) levels in SLE patients. Each data point represents a single subject. Correlation analyses were done using Spearman's rank correlation. sCD226: soluble CD226; SLE: systemic lupus erythematosus.

Supplementary Figure S3

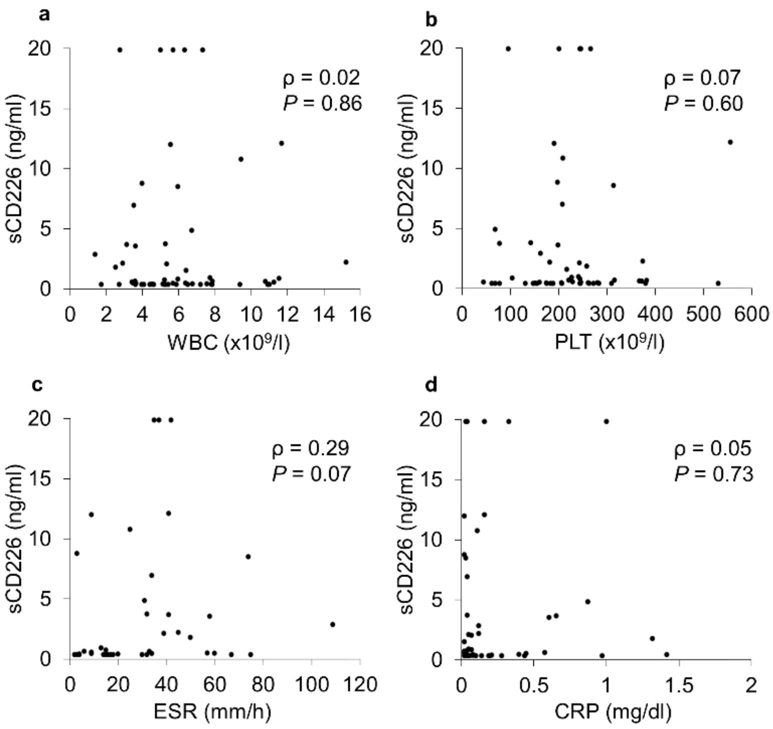

Supplementary Figure S3 Associations between serum sCD226 levels and laboratory findings

Correlations between serum sCD226 levels and white blood cell (WBC) count (a), platelet (PLT) count (b), erythrocyte sedimentation rate (ESR) (c), and C-reactive protein (CRP) levels (d) in SLE patients. Each data point represents a single subject. Correlation analyses were done using Spearman's rank correlation. sCD226: soluble CD226; SLE: systemic lupus erythematosus.

Supplementary Table S1 Serum sCD226 levels in SLE patients with clinical SLEDAI-2K descriptor

SLEDAI-2K descriptors		median sCD226 levels [IQR], ng/ml		*P*-value
	n (%)	presence	absence	
Seizure	1 (2)	0.12	0.31 [0.10–3.42]	0.59
Psychosis	3 (5)	8.68 [4.69–12.0]	0.24 [0.10–1.99]	0.03
Organic brain syndrome	1 (2)	0.15	0.31 [0.10–3.42]	0.70
Visual disturbance	2 (3)	16.0 [12.0–20.0]	0.26 [0.10–2.48]	0.04
Cranial nerve disorder	1 (2)	12.0	0.29 [0.10–3.00]	0.18
Lupus headache	5 (9)	1.29 [0.43–4.98]	0.22 [0.10–3.42]	0.22
Cerebrovascular accident	0			
Vasculitis	0			
Arthritis	11 (19)	2.65 [0.20–12.0]	0.22 [0.10–1.84]	0.02
Myositis	3 (5)	20.0 [20.0–20.0]	0.24 [0.10–1.99]	0.01
Urinary casts	17 (29)	0.36 [0.10–4.12]	0.29 [0.10–2.32]	0.90
Hematuria	15 (26)	0.38 [0.12–3.55]	0.29 [0.10–2.65]	0.33
Proteinuria	20 (34)	0.89 [0.10–7.48]	0.21 [0.10–1.88]	0.25
Pyuria	11 (19)	3.49 [0.10–10.7]	0.22 [0.10–1.90]	0.12
Rash	19 (33)	1.56 [0.20–8.68]	0.16 [0.10–1.84]	0.02
Alopecia	6 (10)	0.39 [0.20–7.66]	0.26 [0.10–3.18]	0.54
Mucosal ulcers	7 (12)	6.82 [0.49–20.0]	0.22 [0.10–1.99]	0.02
Pleurisy	3 (5)	0.22 [0.10–3.49]	0.31 [0.10–3.36]	0.79
Pericarditis	4 (7)	2.05 [0.23–15.9]	0.26 [0.10–2.83]	0.40
Fever	6 (10)	3.42 [1.20–14.0]	0.23 [0.10–1.96]	0.08

Serum sCD226 levels were compared between SLE patients with each clinical SLEDAI-2K descriptor and those without. Statistical differences among groups were evaluated using the Mann–Whitney *U* test. sCD226: soluble CD226; SLE: systemic lupus erythematosus; SLEDAI-2K: SLE Disease Activity Index 2000; IQR: interquartile range.
